# Reproducibility of AI-Informed Coronary Computed Tomography Angiography–Derived Coronary Plaque Volume Quantification

**DOI:** 10.1016/j.jacadv.2026.103001

**Published:** 2026-07-11

**Authors:** Dae-Won Kim, Biyanka Jaltotage, Tim Fonte, Nicholas Ng, Jonathon Leipsic, Georgios Tzimas, Bethlehem Mengesha

**Affiliations:** a University of British Columbia, Vancouver, British Columbia, Canada; b Department of Cardiology, Fiona Stanley Hospital, Perth, Australia; c Heartflow.inc, San Francisco, California, USA; d Heartflow.inc, Mountain View, California, USA; e CentreHospitalier Universitaire Vaudois (CHUV), Lausanne, Switzerland

**Keywords:** AI-CPA, atherosclerosis, CCTA, coronary artery disease, plaque quantification

Accurate, reproducible quantification of coronary plaque volume derived from coronary computed tomography angiography (CCTA) is creating an opportunity to monitor disease progression and assessing the effectiveness of therapeutic interventions.^1^ Variability in measurements, potentially stemming from different analysis software versions, scanner models, or analyst interpretation, can significantly impact clinical decision-making and trial results.

The aim of the study was to assess the interobserver and intraobserver reproducibility of artificial intelligence (AI)-informed CCTA-derived coronary plaque volume quantification (AI-CPA) for the quantifying of total plaque volume (TPV) and plaque composition.

The majority of participants were enrolled from an on-label, post-market, multicenter, retrospective registry, REFINE registry (25/27), while the other 2 were enrolled from a Heartflow commercial population from 2 academic medical centers. All sites provided local institutional approval to analyze and publish any metadata available from the AI-CPA. Statistical analysis was performed using SAS software, version 9.4 (SAS Institute). Intraobserver and interobserver variation of the next-generation AI-CPA (Heartflow)^2^ was assessed in 30 CCTAs selected to represent a broad variety of plaque burden, image quality, and CT vendors. Each study was analyzed 3 times by 3 operators, all trained and certified in the Heartflow analysis workflow, in a blinded manner. Three analyses were excluded due to incomplete data, as TPV across all vessels could not be determined because of stents or imaging artifacts.

A total of 27 patients were included in the study, the mean age was 61 ± 10 years, males accounted for 63%, and the mean body mass index was 27.8 ± 4.6 kg/m^2^. Clinical variables were available among 25 of the study participants, where 16/25 (64%) had dyslipidemia, 15/25 (60%) had hypertension, and 4/25 (16%) had diabetes mellitus. Prior and current smoking history was reported among 11/25 (44%) and 4/25 (16%), respectively.

There was variability in the type of CT scanners used across centers. SIEMENS scanners were the most commonly used 15/27 (55.6%), including Siemens SOMATOM Definition AS 11.1%, Siemens SOMATOM Definition Flash AX 40.7%, and Siemens SOMATOM Force 3.7%. GE MEDICAL SYSTEMS scanners accounted for 7/27 (26%), including GE Revolution 22% and GE Revolution Apex 3.7%. Philips iCT 256 scanners represented 5/27 (18.5%) of the included studies.

Among 25 patients for whom data on demographics and medical history were available, 16/25 (64%) were found to have obstructive coronary artery disease (≥50% stenosis) based on Roadmap analysis (AI-driven coronary stenosis detection) on CCTA. Among these, 9/25 (36%) had moderate stenosis (50% to 69%), 6/25 (24%) had severe stenosis (≥70%), and 1/25 (4%) had total occlusion.

Plaque volume was computed and compared across the 243 independent AI-CPA analyses. The total number of independent plaque volume measurements (n = 243) was derived as the product of the total measurements in the 3 coronary arteries for the entire study population (81 analysis) across the 3 operators (81 x 3 = 243). The analysis was mixed model with observer and patient as random effect. The median values and IQR of plaque volumes were as follows: TPV 295 mm^3^ (IQR: 137-450 mm^3^), calcified plaque volume (CPV) 25 mm^3^ (IQR: 3-112 mm^3^), noncalcified plaque volume (NCPV) 238 mm^3^ (IQR: 124-384 mm3), and low attenuation plaque volume (LAPV) 0 (IQR: 0-0). The coefficient of variation was used to compare relative variability across measurements. The interobserver variability for TPV, CPV, and NCPV were 3.1% (95% CI: 1.6%–9.6%), 5.7% (95% CI: 4.0%-15.4%), and 3.9% (95% CI: 2.5%-9.0%), respectively, indicating excellent consistency between readers. Similarly, the intraobserver variability for the quantification of TPV, CPV, and NCPV were 5.3% (95% CI: 4.7%–5.9%), 6.4% (95% CI: 5.8%–7.2%), and 5.9% (95% CI: 5.3%–6.6%), respectively, suggesting excellent intrareader reproducibility. Both interobserver and intraobserver reproducibility were modest for LAPV. This was likely attributed to the low quantity of LAPV measured and the limited number of individuals with LAP. Fleiss' kappa was assessed for agreement of the 243 analyses to categorize patients into TPV plaque stages (0, 1-100, 101-250, 251-750, ≥751 mm^3^); the resulting kappa value was 0.95 (95% CI: 0.91-0.99).

The mean-SD plot showed that measurement variability between readers increased with higher mean plaque volumes, with lower variability observed at lower plaque volumes, particularly for TPV, CPV, and NCPV (Figure 1).

**Figure 1:**
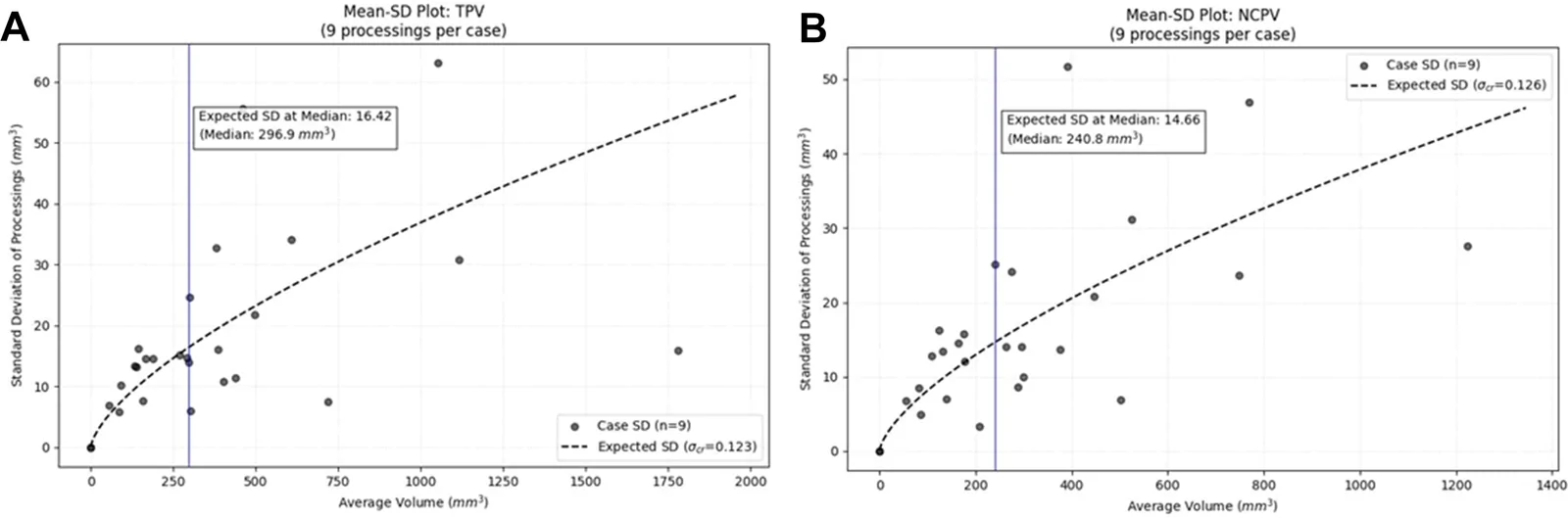
Plaque volume variability for TPV and NCPV Mean-SD plot for total plaque volume (A) and noncalcified plaque volume (B). Each point on the plots represents a patient, with the x-axis representing the mean volume for each patient and the y-axis representing the SD for each patient. The dashed line represents the expected SD based on assumption that variability is constant in the cube-root space. NCPV = noncalcified plaque volume; TPV = total plaque volume.

AI-CPA has shown high agreement with intravascular ultrasound for the quantification of plaque volume and plaque characterization.^3^ A recent scientific statement by the American College of Cardiology on AI-CPA recommends the use of AI-CPA in patients with visual evidence of plaque on CCTA to enhance cardiovascular risk assessment and guide the initiation or intensification of preventive therapies.^4^ However, robust reproducibility of AI-based plaque quantification is required to ensure clinical applicability. The 2025 American College of Cardiology scientific statement recognizes the need for vendors to develop standardized AI-CPA algorithms that account for differences in detector type, kVp, and reconstruction parameters.^4^ The recommendations by the scientific statement in AI-CPA reporting include specifying the name of the software/vendor used and a statement on reproducibility data of the AI-CPA software,^4^ further highlighting the need for each software/vendor to study and report its own reproducibility. Iraqi et al have demonstrated interscan reproducibility of plaque volume measurements using standardized semiautomated software for quantitative plaque assessment.^5^ Our study builds upon existing literature by demonstrating the high interobserver and intraobserver reproducibility for TPV as well as plaque components (NCPV and CPV) using AI-CPA, regardless of variability in CT vendor type and image acquisition.

Our study has several limitations including the variability in scanner and image acquisition across the study cohort. However, given the study’s main objective was interobserver reproducibility rather than interscan agreement, the limitation has less impact in the main conclusion of the study. Regardless, the robustness in AI-CPA reproducibility held true despite the significant heterogeneity in the imaging acquisition techniques and parameters in the study cohort.

Overall, our study shows excellent interreader and intrareader agreement and consistency in ranking between the measurements obtained by the 3 independent analyses. There was minimal interreader and intrareader variability in the TPV, CPV, and NCPV measurements obtained by the 3 independent analyses. These findings are promising for the application of quantitative plaque analysis in serial follow-up imaging, both in clinical practice and in future trials.

## Funding support and author disclosures

Dr Leipsic has a relationship with HeartFlow Inc that includes consulting or advisory and equity or stocks. Mr Fonte has a relationship with HeartFlow Inc that includes employee and equity. Mr Ng has a relationship with HeartFlow Inc that includes employment. All other authors have reported that they have no relationships relevant to the contents of this paper to disclose.

**What is the clinical question being addressed?**

In this Gage R&R study, we demonstrated high interobserver and intraobserver reproducibility for quantification of TPV as well as plaque components NCPV and CPV using AI-CPA, regardless of variability in CT vendor type and image acquisition.

**What are the main findings?**

Reproducibility of LAPV measurement was modest and was likely attributed to the low quantity of LAPV measured and the limited number of individuals with LAP.
